# Tourism during health disasters: Exploring the role of health system quality, transport infrastructure, and environmental expenditures in the revival of the global tourism industry

**DOI:** 10.1371/journal.pone.0290252

**Published:** 2023-09-06

**Authors:** Yu Xiong, Xiaohan Tang

**Affiliations:** 1 School of Environmental and Chemical Engineering, Foshan University, Foshan, 528000, Guangdong, China; 2 Decision Consulting Department, Party School of the Zhongshan Municipal Committee of the Communist Party of China, Zhongshan, 528403, Guangdong, China; University of City Island, CYPRUS

## Abstract

Tourism is one of the most important promoters of sustainable development in many nations and regions around the globe. Tourism expansion has been a significant contributor to social and economic growth, particularly in developing economies. It is, however, vulnerable to all kinds of health crises and natural disasters, no matter how small they are. The primary purpose of this study is to acquire an empirical understanding of the effects of health crises and disasters on international tourism. The SYS-GMM was used to examine the impact of health calamities and crises, carbon footprints from transportation, and green finance on the tourism of 51 countries between 2007 and 2020. The results showed that health crises and natural disasters have a big effect on international tourism. Alternatively, the presence of eco-friendly and secure transportation at tourist destinations has a positive effect on the tourism industry. The results also showed that environmental expenditures have positive short- and long-term effects on international tourism. Furthermore, the sensitivity of travelers to health crises and natural disasters varies over the short and long term. The study also showed that compared to normal times, international tourism dropped by more than 67% during COVID-19. Consequently, this research assists us in comprehending, predicting, and preventing the potential adverse effects of COVID-19 and other similar economic, health disasters and crises that could occur in the future and harm the tourism industry.

## 1. Introduction

The tourism industry has evolved rapidly over the last three decades in several countries around the globe, and it has been widely recognized as a key driver of economic growth and development, especially in emerging markets and developing countries. Tourism is also one of the most important sectors owing to its large contribution to global employment [1,2] but different crises, both natural and human-induced, often disrupt such development. These crises not only pose a threat to the tourism sector, but they also have a significant impact on traveler spending and behavior [3]. For example, the SARS health crisis in 2003 adversely affected the tourism sector in East Asia. The Ebola outbreak in 2014 significantly reduced tourism growth in West Africa. Similarly, the ongoing Syrian and Ukraine wars not only affected the tourism industry inside these countries but also in surrounding regions. Acts of terrorism and political instability in different parts of the world also negatively affect the global tourism industry [4–7].

Even though the tourism industry has become more resilient to various crises, it is still the sector most affected by the COVID-19 pandemic. The current health crisis confronting this industry is not only distinct from previous ones, but it may also have significant long-term implications for tourism as a socioeconomic activity and industry. The most important things about this pandemic are its global and huge size, as well as its many and interconnected effects, which challenge current values and systems and cause a global economic slowdown and depression [8,9]. The COVID-19 pandemic is wreaking havoc on the world’s political, social, and economic systems. All of these things can affect the attitudes and behaviors of tourists about the selection of a tourism destination in the new normal. The global tourism industry has been negatively impacted by public health and social strategies (social distancing, mobility restrictions, COVID-19 testing and quarantine requirements, prohibiting mass gatherings, and smart lockdowns) [3,10].

The COVID-19 will have different impacts on the tourism industry of various countries due to its uneven economic impacts and depending on the strictness of public health and social measures (closing borders, imposing isolation requirements, and reducing transportation) [11]. Global tourist mobility was expected to decline by 80%, costing the global economy more than $1.2 trillion in 2020. More than 120 million tourism workers have lost their livelihoods due to COVID-19, showing the profound impacts and fragility of the tourism sector in the face of this health crisis [12,13].

Crises may bring about change, but the tourist industry has not yet undergone a profound transformation [14]. Crisis-led reforms will depend on how these stakeholders are affected by crises, react to, recover from, and imitate on crises. The growing implications of the present health crisis on the tourism sector have sparked widespread interest among academics and policymakers alike in exploring and seeking strategies to expand and sustain the sector during the pandemic. Everyone agrees that this pandemic should be seen and used as a chance for transformation in the tourism sector [15]. Along with recovery, the tourism sector should rethink and remodel itself for the new normal [16]. A number of previous studies examined the impact of COVID-19, but these do not expand our understanding or direct the industry in a new direction. Moreover, COVID-19 will have significant effects on economic, psychological, and environmental factors that all affect each other, we can expect unexpected trajectory changes rather than historical patterns, and traditional predictors may not be accurate enough. Therefore, in order to reveal the true impact of COVID-19 on tourism, studies should also consider the other important predictors along with COVID-19, and some researchers [17–19] have already started putting in efforts to explore the factors that can help to sustain this fragile industry during the health crises. The prior literature also recommends that tourism COVID-19 research should dig deeper into the causes and responses of stakeholders to COVID-19’s effects in order to better understand, predict, educate, and bring about change in the tourism industry. Otherwise, we would just keep having crises after crises [20]. The tourism research should also not only consider the experiences of stakeholders in various countries with health crises but also other related issues such as green finance.

Green financing is recognized as one of the pathways to foster sustainable economic growth and achieve several of the current United Nations’ sustainable development goals. Environmental quality is prioritized more in green finance than in traditional finance, which places less emphasis on ecological conservation and efficient resource use. Green finance targets to attain sustainable development by incorporating financial activities with ecological protection. Green finance is a cutting-edge financial concept that combines ecological protection with financial benefits [21]. A very scarce body of literature [22,23] is available on the impact of green finance on the tourism industry. None of the studies assessed the impacts of green finance along with the implications of COVID-19 and other health crises on the world tourism industry.

Therefore, the following research questions have been formulated: i) To what extent does a health disaster or crisis impact tourism at a destination? ii) To what extent does the quality of health services contribute to the revitalization of the tourism industry? iii) To what extent does green finance affect the tourism industry? To what extent do air quality and transportation carbon footprints affect global tourism? Based on the aforementioned research inquiries, it is possible to construct research hypotheses; for instance, the anticipated health disaster or crisis is projected to have an adverse impact on the international tourism industry. The quality of health services and implementation of green finance are anticipated to have a positive impact on the revitalization of global tourism. The adverse impacts of suboptimal air quality and inadequate transport infrastructure significantly undermine the global tourism industry.

So, the main goal of this study is to look at the effects of current and past health crises, as well as green financing, on the global tourism industry. This will help professionals and academics better understand and value both the touristic effects and transformative opportunities of health crises and green financing, as well as filling in gaps in the literature. The first objective of this research was to analyze the effects of health disasters and crises on international tourism. The second objective of the study was to explore the effect of green financing on the world tourism industry. Another key objective of this study was to examine the impact of transportation carbon footprints and safe transportation system on the tourism inflows.

This study is the first to examine the impacts of health disasters and crises, air quality, and green financing on the global tourism industry. It would contribute significantly to decision-making on sustainable tourism during the ongoing health crisis, as well as how transitioning to a green economy can affect international tourism.

This research is an important addition to the growing body of scientific literature. It will help us understand, predict, and prevent the possible negative effects of the COVID-19 crisis and other similar economic and health crises that could happen again in the future and hurt the tourism industry. Furthermore, this research will improve our understanding of how to support, encourage, shape, or even lead the tourism industry during health crises and disasters.

## 2. Review of literature

The term "disaster" refers to an occurrence characterized by a sudden, unpredictable, or significant manifestation of unfortunate circumstances [24]. Carter [25] postulated that a disaster can be classified as either natural or man-made, characterized by its abrupt or gradual occurrence, resulting in profound consequences for society as a whole in that affected economies must implement unusual measures. Moreover, disasters are defined as "a significant disturbance in the operations of an entire society or community that leads to extensive material, human, economic, or ecological harm that surpasses the affected community’s or society’s capacity to manage through its own natural resources"[26]. According to the World Health Organization [27], a health crisis is characterized as an occurrence that surpasses the ability of a community to manage. In the context of this discussion, it pertains specifically to the emergence of an infectious disease epidemic. Therefore, disasters that transpire to tourist destinations have a profound and detrimental impact on the local tourism industry. In the present study, a disaster is defined as an occurrence, whether natural or man-made, that transpires abruptly or gradually and results in severe harm or injury, leading to loss of life.

Attractions are considered essential components of tourism [28]; diverse cultures and environments exert a magnetic pull on tourists seeking respite in their everyday routines. However, the fifth "s," which stands for security, may be more important than the other four seconds that draw people in sand, sea, sun, and sex. In contemporary times, the increasing perception of vulnerability arising from acts of terrorism and natural disasters has become a significant factor in shaping the appeal of a destination for tourism [29]. Consequently, the level of assurance provided to tourists regarding their safety and security has emerged as a crucial determinant for evaluating the attractiveness of such destinations [30]. Travel behavior, which is a form of consumer behavior, is inherently associated with various risks. When individuals actively pursue activities or experiences that provide excitement, there is a potential for risk that could compromise their security and lead to undesirable outcomes. Consequently, destinations perceived as hazardous or insecure may be dropped as alternatives [31].

The occurrence of sudden insecurity has a notable influence on the demand for tourism [32] as it is linked to instances of disaster, loss of life, and tragic events. There is a perception among certain tourists that disasters can happen unpredictably and in various forms, leading to fear of engaging in leisure activities [33]. Tourism possesses a distinctive characteristic whereby its various activities are susceptible to risks that pose threats to both health and security [30]. These disasters can be perceived as significant challenges to tourist attractions, as tourists frequently exhibit a clear inclination towards a peaceful social environment [34,35].

The preservation and maintenance of the environment, encompassing both natural and man-made elements, play a crucial role in the development and sustainability of tourism. Nevertheless, the interplay between tourism and the environment is multi-faceted. This encompasses a multitude of activities that have the potential to have detrimental effects on the environment. Numerous consequences are associated with the development of essential infrastructure, such as transportation networks and aviation hubs, as well as the establishment of tourism services encompassing resorts, hotels, dining establishments, retail outlets, golf courses, and marinas. Air quality plays a significant role in tourists’ decisions [36]. The number of incoming tourist arrivals in China has exhibited a downward trend in recent years, potentially because of rising levels of urban air pollution. According to Becken et al. [15], the perception of air quality risk has a notable adverse influence on both the image of the destination and the decision to visit China.

Potential tourists exhibit a keen interest in places to visit that possess both aesthetic appeal and cleanliness, while also providing services of exceptional quality. Nevertheless, an environment lacking the characteristics of a high-quality environment not only fails to attract visitors, but also fails to provide an enjoyable environment for the residents of the area [37]. According to Shaalan [38], the deterioration of distinct and natural habitats in numerous emerging economies has led to the loss of their significant competitive edge. There is a growing trend among tourists to exhibit greater discernment in their choice of destination. One significant factor that has gained prominence in the decision-making process is the environmental conditions of potential destinations. According to Kastenholz [39], sustainable tourism necessitates the implementation of effective tourism demand administration strategies. Enhancing regional social and cultural awareness of tourists and promoting responsible behavior towards the environment are crucial factors that may contribute to the overall competitiveness of destinations.

In summary, attractions play a significant role in the advancement of tourism. However, relying solely on attractions as a prerequisite for the glory of a tourist destination is insufficient. This is due to the fact that the absence of safety measures, low air quality, poor infrastructure, and dirty environment can hinder the growth and sustainability of tourism, as emphasized by the statement "no safety, no tourism" [33].

## 3. Materials and methods

### 3.1. Data sources and variable measures

The empirical analysis of the current study is based on a time-series dataset for both developing and developed countries. Due to a lack of required data, the number of countries considered has been reduced to 51 in this study. The variables of the study comprise tourism, health crises, transport infrastructure, healthy system quality, environment, and green finance-related factors that affected the tourism industry over the period of 2007–2020. The current study incorporates data from a variety of sources, such as the World Development Indicators (WDI), the International Monetary Fund (IMF), and the World Health Organization (WHO).

#### 3.1.1. Tourism measure

A country’s tourism can be measured using a variety of measures, including the amount of money spent on leisure travel, business travel, travel and tourism that directly contributes to GDP, and the number of tourists who cross its borders. The current study considered international tourism receipts (US dollars) to capture the tourism.

#### 3.1.2. Health crises and disaster measure

A crisis denotes "occurrences that are more tightly related to the company’s surroundings," but a disaster, on the other hand, is the outcome of an external action that may have an immediate impact [40]. Therefore, the event that suddenly causes an unfavorable situation is called a "crisis" [41]. Both disasters and crises significantly affect the tourism industry. Their effect on tourism may be in the form of reduced tourist flows, increased unemployment, and decreased investment in the sector [42]. Moreover, peace, safety, and security are prerequisites for revolutionizing the international tourism industry, and health crises and disasters significantly affect all of these variables, which alter behavior and normal tourism trends worldwide [43]. The fact that the number of deaths in a country that is a tourist destination is going up shows that the country is not safe for tourists to visit. For example, as dengue fever caused by mosquitoes re-emerged as a major public health challenge worldwide, it caused serious health problems and raised mortality rates [44,45]. Similarly, the current pandemic has paralyzed global health systems, resulting in a massive increase in mortality rates in visited countries. European countries are among the top tourist destinations, but the mortality rate in this region is higher than in any other region of the world, making it dangerous for tourism inflow. Thus, the present health crisis has not only increased mortality but also created mental and health issues for tourists [46–48]. Therefore, the number of fatalities indicates how serious the health concerns are at any tourist destination [42]. The number of deaths in tourism destinations each year was considered in this research to assess the effect of health crises on the countries’ tourism industries.

#### 3.1.3. Transport infrastructure quality measure

Tourist road injuries and deaths are a major concern for tourist authorities and policymakers working to increase tourism in their respective countries. A tourist with a bad experience will share their negative story with an average of 11 people. Wilks et al. [49] describe word-of-mouth referrals as one of the most important sources of information in making a holiday decision. Moreover, transitioning towards eco-friendly transport is essential for a sustainable tourism industry in any country. Azimi [50] stated that safe, cheap, and eco-friendly travel is a prerequisite for tourism development. Therefore, this study considers the deaths from road injuries in accidents, and carbon footprints of transport as a measure of transport infrastructure quality at tourist destinations.

#### 3.1.4. Health system quality measure

Health spending per person is affected by a wide range of social and economic factors, as well as the organizational structure of a country’s health system [51]. As a result, this study looked at health expenditures per capita as a proxy for a country’s health-care system’s quality.

#### 3.1.5. Air quality measure

Air quality is also a very important part of tourism, since the main reason people travel is to get away from their everyday lives and find a place where they can relax and release stress [52]. So the air quality must be considered a prime determinant in the tourists’ decision-making process because it may significantly affect the competitiveness of the tourism destination [53,54]. Moreover, the air quality is highly associated with a health risk [55]; it affects the physical comfort, which is very important for the tourists’ experience and their health. Seaton et al. [56] and Eusébiov et al. [57] described that short- and long-term exposure to ambient air pollution can cause a wide variety of chronic and acute health issues. Similarly, the air quality also causes aesthetic enjoyment, which is highly affected by the presence of particles and haze [58]. This study used PM2.5 (micrograms per cubic meter) as a measure of air quality in tourist destinations.

#### 3.1.6. Green finance measure

As a measure of green finance, this study considers a touristic country’s environmental expenditure for reducing the types of pollution (environmental protection research and development, pollution abatement, and waste management) and investment for ecological conservation (protection of biodiversity and landscape).

### 3.2. Control variables

This study has used two control variables (per capita income and population), as these variables are likely to affect the size of the response to government economic and tourism policies. The per capita income significantly differs among countries, which affects the development of tourism policies [59]. Furthermore, tourist flow is strongly related to the destination country’s population and per capita income because tourists expect not only cultural and natural resources but also the best service related to business and leisure, such as restaurants and infrastructure. Therefore, the richer and bigger a country is, the greater will be its supply of tourist services [60].

### 3.3. Empirical model

This research used the system generalized method of moments (SYS-GMM) model to analyze the effect of health disasters and crisis, transport infrastructure quality, air quality, the health system, and green finance on international tourism. This model was preferred for the present data set because it can address many hidden problems in the panel data such as endogeneity, correlation, heteroskedasticity, and autocorrelation [61,62]. The disparity between the difference and the system-generalized method of moments (GMM) is common when circumstances are characterized by the following features: a dataset with panels has:1) a relatively small number of time periods (T) and a large number of individuals (N); 2) a linear functional relationship among the variables; 3) a dynamic dependent variable, which means it depends on its own previous values; 4) independent variables that are correlated with past and possibly current error terms, which means they are not strictly exogenous; 5) fixed effects that are specific to each individual; and 6) heteroskedasticity and a nonlinear relationship (autocorrelation) between the individual [63]. The generalized method of moments (GMM) estimator exhibits consistency, indicating that it converges in probability to the true parameter as the sample size approaches infinity, given the appropriate conditions [64]. Similar to the two-stage least squares (2SLS) method, this approach generally exhibits bias because, in infinite samples, the instruments employed are typically at least marginally correlated with the endogenous elements of the instrumented regressors. According to Roodman [63], it is commonly observed that correlation coefficients between finite samples of variables that are initially uncorrelated tend to deviate from an exact value of zero.

Moreover, in cases where the number of time periods is limited and the time series exhibits persistence, it is expected that the GMM estimators will exhibit poor performance after applying the first difference technique [65]. The inadequacy of the lag levels in the series is attributed to their limited utility in the context of differenced equations. The utilization of System GMM is highly recommended because of its demonstrated effectiveness in mitigating bias arising from measurement error, unobserved heterogeneity, variable omission, and the prevalent issue of endogeneity, which often impacts the dependent variable [65–67]. The GMM estimation method has garnered significant attention in empirical research. The System Generalized Method of Moments (GMM) is known for its robustness, efficiency, and ability to handle serious problems, such as autocorrelation and heteroscedasticity in panel data [63]. Furthermore, the applicability of fixed effect and random effect panel data models is limited when the number of cross-sections (N) exceeds the number of time periods (T), and the time period is relatively small. In an effort to address potential sources of bias arising from endogeneity, missing data, and measurement errors, this study utilized the System-GMM estimate [65,67]. Hence, the presence of endogeneity resulting from country-specific effects [68] and the utilization of data from dynamic panels with a time period of 14 and a large sample size of 51 countries [69,70] necessitate the use of the generalized method of moments estimators (GMM) in this research.

The GMM approach proposed by Arellano and Bover [67] and Blundell and Bond [65] considers the lagged values of the dependent variable as an instrument to tackle the endogeneity problem of panel data. To account for the presence of a lagged endogenous variable while also accommodating for potential endogeneity in the remaining explanatory variables [70]. The GMM estimator is utilized in this study to address the issue of country-specific impact by employing all possible lagged levels as instruments after applying first differencing. The System GMM estimator proposed by Blundell and Bond [65] and Arellano and Bover [67] combines a set of moment conditions derived from the equation in levels with the conventional set of moment conditions in first differences, utilizing lagged levels as instruments [65,67].

In our GMM estimation, we used Arellano and Bover’s (67) specification tests to determine how well the instruments worked as an empirical inquiry [67]. The validity of the additional moment constraint necessary for the system GMM is assessed using Hansen’s statistics. These tests were employed to examine the null hypotheses pertaining to instrument validity. The validity of the instrument was established if the null hypothesis was not rejected. Additionally, the Arellano-Bond test was employed to examine the presence of second-order serial correlation in the first-differenced residuals. The null hypothesis posits that residuals exhibit serial uncorrelations. If the null hypothesis cannot be rejected, it suggests that there is no second-order serial correlation and that the generalized method of moments (GMM) estimator is reliable.

Even though both types of GMM models, such as difference GMM and system GMM, have gained popularity among research scholars, Levine et al. [71] describe that the efficiency of the difference GMM decreases in the case of a small sample size and that it can generate biased estimates if the data are not stationary. While using the same data, the SYS-GMM can provide more accurate and unbiased estimates [72]. Additionally, when the time series is a random walk process, the SYS-GMM is better because it also provides efficient estimates for the endogenous variables [65]. Moreover, between the two types of GMM, Diff-GMM employs moment conditions to determine the first difference, thereby eliminating fixed effects. In contrast, Sys-GMM integrates the moment conditions and conducts simultaneous estimations of both differences and levels [63,73]. The use of the system generalized method of moments (sys-GMM) offers several advantages over the difference generalized method of moments (diff-GMM) approach. These advantages are particularly evident in panel data settings, where the number of cross-sectional units (n) exceeds the number of time periods (t). The sys-GMM method is more suitable when a linear function exists in the model, the dependent variable exhibits dynamic behavior, the explanatory variables are not completely exogenous, unknown heterogeneity is present, autocorrelation is present within the variables but not across them, and heteroscedasticity exists in the data. Specifically, sys-GMM exhibits a smaller bias and root mean squared errors [74]. Additionally, Yamarik and Redmon [75] found that sys-GMM offers a higher level of accuracy relative to diff-GMM in cases where the dependent variable displays persistence. One additional benefit of employing the system generalized method of moments (sys-GMM) is the ability to utilize internal instruments to identify endogenous variables [76,77]. In conclusion, the system generalized method of moments (sys-GMM) demonstrates superior estimation accuracy compared with the difference generalized method of moments (diff-GMM). This validates the preference for employing the sys-GMM over the diff-GMM.

The extended version of the GMM estimator, also known as the System-GMM estimator developed by Blundell and Bond (65), is considered acceptable and has been employed in this study because of its evident advantages over other methods used to estimate standard linear dynamic panel data models [65]. Keeping in view the advantages of SYS-GMM, this study used the following SYS-GMM model to determine the estimates for tourism.


$$y_{it}=\lambda y_{it-1}+\beta x_{it}+\gamma z_{it}+\eta_{i}+\varepsilon_{it}$$

$$u_{it}=\eta_{i}+\varepsilon_{it}$$

$$\eta_{i}∽IID(0,\sigma_{\eta }{}^{2}),\;\varepsilon_{it}∽IID(0,\sigma_{\varepsilon }{}^{2})\;\mathrm{and}\;E[\eta_{i}\varepsilon_{it}]=0$$

y_it_ (dependent variable) describes the tourism receipts, which describe the revenue received from tourist inflows in a country; y_it_-1 represents its lagged value. The z_it_ is the vector of control variables; the x_it_ describes the vector of explanatory variables; and the u_it_ is the error term; it includes the "random error term" and the unobserved country-specific effect or fixed effect. A necessary condition of SYS-GMM is that the error term should not have second-order serial correlation; otherwise, the standard error for instrument estimates grows without bound [51]. Therefore, Sargan or Hensen test for testing the over-identification, and Arellano-Bond [AR2] for testing the autocorrelation [42] were employed to ensure the reliability of the data sets in the study.

## 4. Results

### 4.1. Descriptive statistics

The main objective of this study is to analyze the effects of health crises, transport infrastructure quality, and green finance on international tourism. Descriptive statistics for the different variables for the 51 countries are presented in Table 1. The standard deviation is an important parameter that describes the dispersion of the data points around the mean value; a low standard deviation indicates that the values are near the sampled mean. The standard deviation for the tourism variable was 1.28 in the sampled countries, with the mean log value equal to 22.19. It implies that the sampled countries have their tourism receipts between 20.91 (22.19–1.28) and 23.47 (22.19+1.28). Moreover, the mean value is close to the maximum value, indicating that the tourism level is good in the sampled countries. The mean log value of the health expenditures was 6.65, with a 1.45 standard deviation, which implies that all the sampled countries have health expenditures between 5.2 and 8.14. Their average value, which is far from their minimum value, indicates that the countries have good health. The transportation carbon footprint has a mean log value of 3.30 with a standard deviation of 0.50 for tourist destinations. The standard deviation of carbon footprints indicates that countries have carbon footprints in the range of 2.8 and 3.8. Moreover, a mean value closer to the maximum carbon footprint value indicates that the country’s transport is not at a good level. The mean log value of the mortalities from road injuries was 2.35, with a 0.65 standard deviation. Thus, the road injuries in the sampled countries range between 1.7 and 3.0. The mean log of deaths due to health crises was 9.08, with a 2.41 standard deviation. The air quality has a standard deviation of 0.60 and a mean log value of 2.95. The standard deviation of green finance was 2.64, and the mean log value of the sampled tourist countries was 20.18. The standard deviation of deaths due to health crises explained the death rate in the sampled countries, ranging from 6.67 and 11.49. Similarly, the air quality of the sampled farmers ranges between 2.35 and 3.55, and its mean value was close to the minimum value, indicating that the sampled countries’ air quality was at a good level owing to the low level of PM2.5. Finally, green finance in terms of environmental expenditures ranges between 17.54 and 22.82, and its mean value closer to its maximum value describes the good level of green finance in the sampled economies.

Regarding the units of the variables before converting them into log form, the tourism receipts and health expenditure variables were measured in US dollars, and the carbon footprints were measured as a percentage of total fuel combustion. Mortality from road injuries and health crises was expressed in terms of the number of deaths; the air quality parameter was measured in micrograms per cubic meter, and green finance was measured in the form of local currency that was converted first into US$ before taking their log form.

**Table 1:** Descriptive statistics.

Variables	Obs.	Mean	Std. Dev.	Min	Max
**Tourism**					
Tourism receipts	661.00	22.19	1.28	16.76	25.01
**Health system quality**					
Health expenditure	661.00	6.65	1.45	2.48	9.20
**Transport infrastructure quality**					
Transportation carbon footprints	661.00	3.30	0.50	1.71	4.24
Mortality from road injuries	661.00	2.35	0.65	0.74	4.17
**Health disasters and crises**					
Mortality from health crises and disasters	661.00	9.08	2.41	4.20	14.12
**Air quality**					
PM2.5 air quality	661.00	2.95	0.60	1.77	4.61
**Green finance**					
Environmental Expenditures	661.00	20.18	2.64	13.41	25.48

The results presented in Table 2 describe the correlation among the different variables of the study. The variable of green finance has the highest positive correlation with tourism. The negative correlation of tourism with deaths, transportation carbon footprints, air quality, and mortality from road injuries is also notable for further analysis.

**Table 2:** Correlation structure.

	Tourism	Health system quality	Transportation carbon footprints	Mortality from road injuries	Health crises	Air quality	Green finance
Tourism	1						
Health system quality	0.626	1					
Transportation carbon footprints	-0.1105	-0.0392	1				
Mortality from road injuries	-0.4673	-0.6281	-0.0082	1			
Health disasters and crises	-0.1326	-0.5198	-0.0165	0.4652	1		
Air quality	-0.3682	-0.6523	-0.1263	0.5567	0.467	1	
Green finance	0.6889	0.556	-0.1621	-0.5458	-0.0103	-0.457	1

### 4.2. Aggregate correlational linear relationship

**Fig 1:**
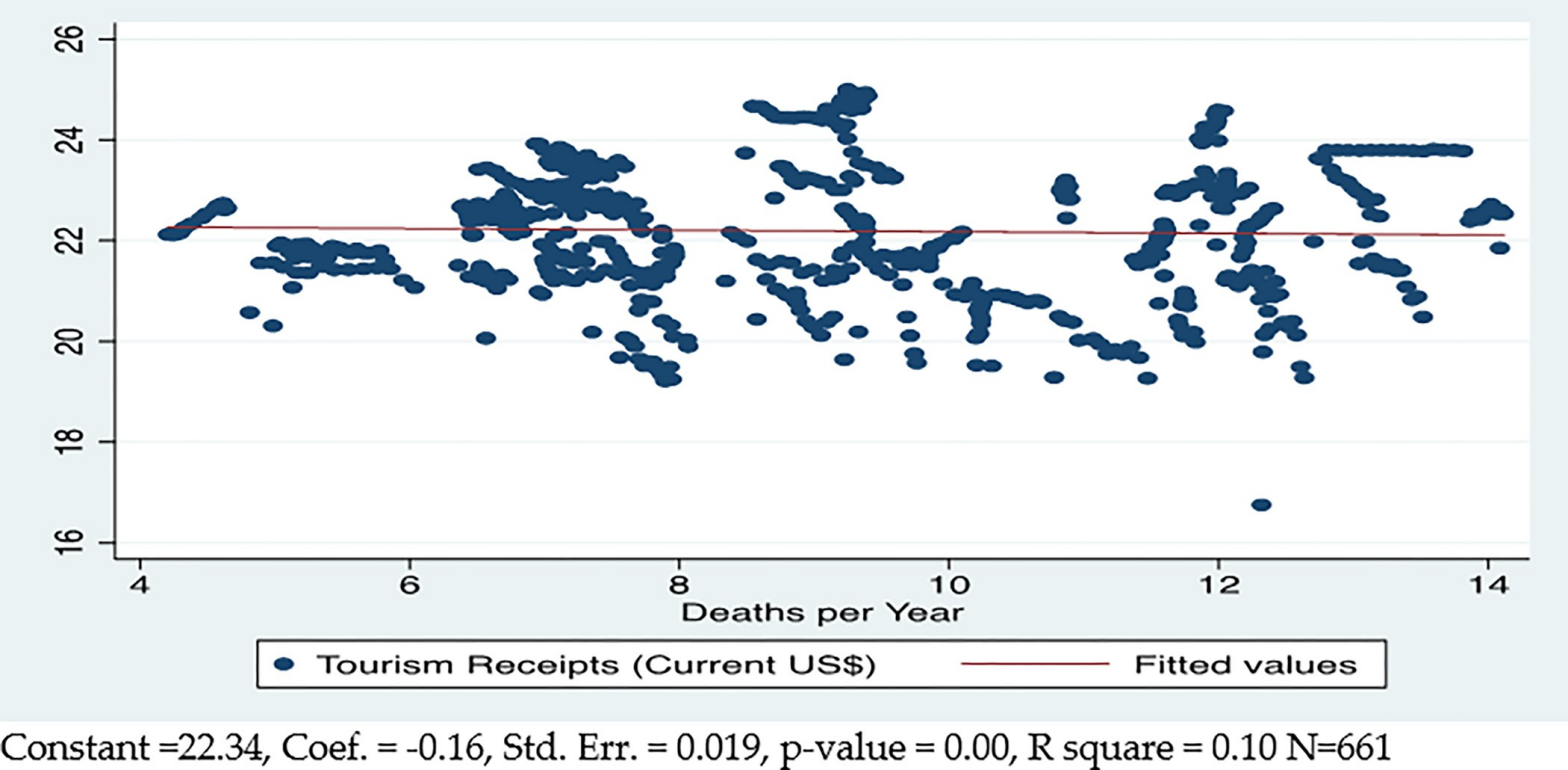
Mortalities due to health crises and disasters and international tourism.

The theoretical literature has suggested positive and negative correlations between the determinants and tourism in this study. The aggregate linear correlation between variables that occurred during per capita income and population changes over time provides useful intuitions. The first aggregate correlational linear relationship between deaths due to health crises and tourism describes significant negative results. This negative relationship between health crises and tourism is shown in Fig 1 for the sampled countries. A 1% rise in deaths in the country due to health crises and disasters is associated with a 0.16% increase in tourism, which is statistically significant at 1%. The deaths due to health crises and disasters explain about 10% of the cross-country variance in tourism.

**Fig 2:**
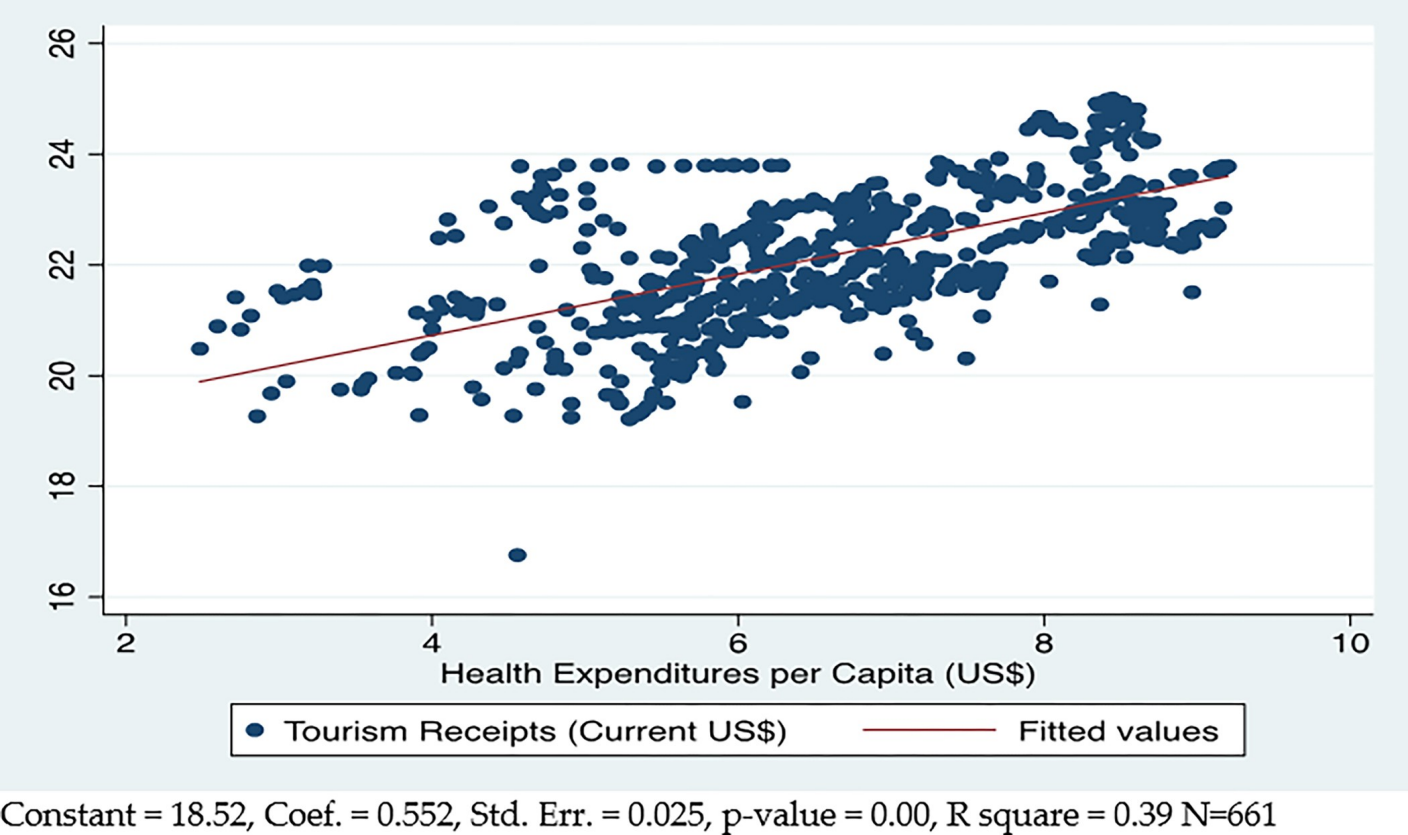
Health system quality and tourism.

Fig 2 explains the positive and significant aggregate correlational linear relation between health system quality and international tourism. The relationship between health systems and international tourism demonstrates that better health systems will increase tourist inflows to those countries. The current results show that a 1% increase in health expenditures caused a 0.55% increase in tourism across the sampled countries. Health system quality explains the 39% variance in tourism across the countries.

**Fig 3:**
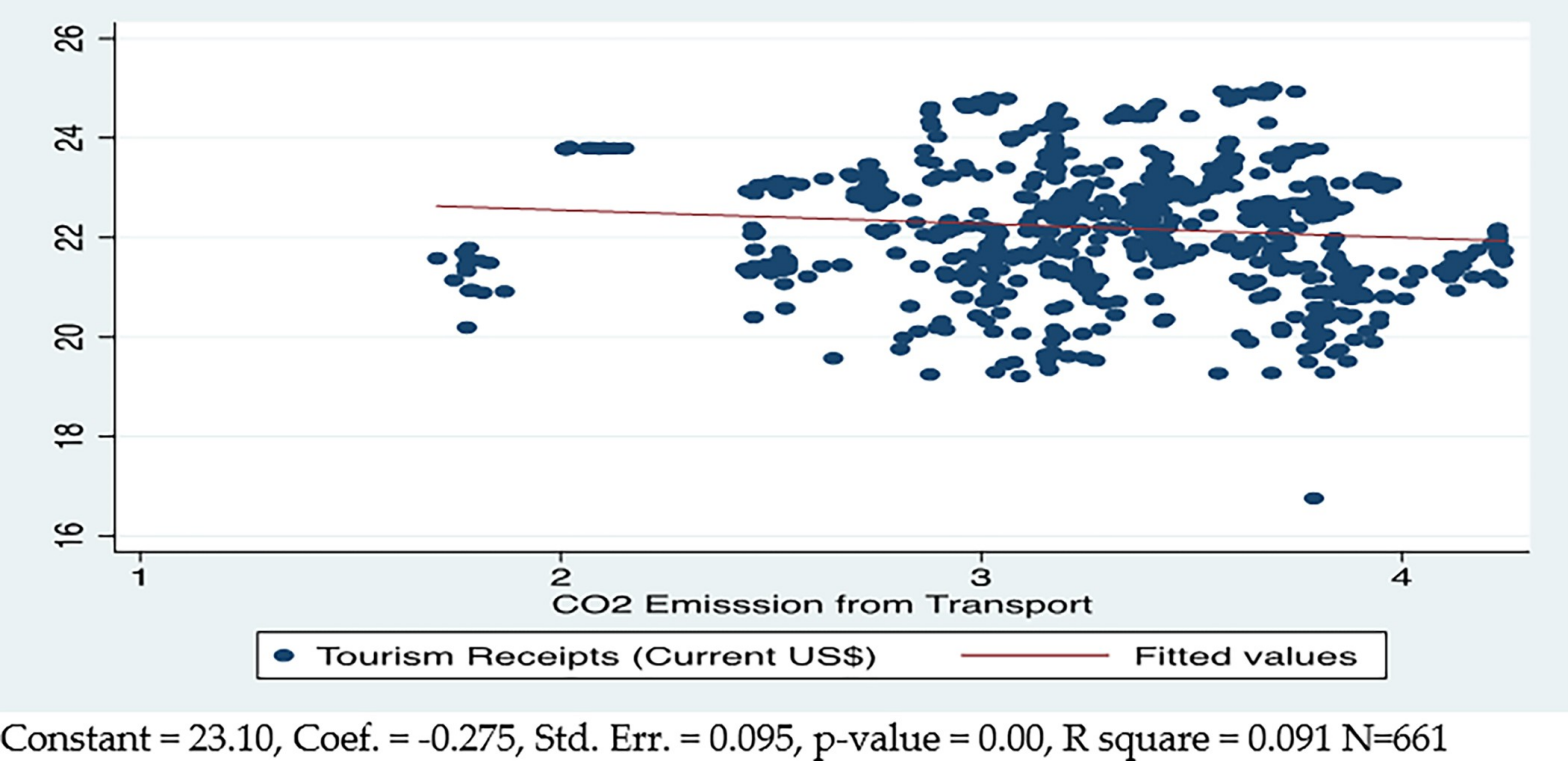
Transportation carbon footprints and tourism.

Fig 3 explains the aggregate correlational linear relationship between transportation carbon footprints and international tourism. The results explain that the 1% increase in CO2 emissions from transport will decrease tourism by 0.27%. It explains the 9.1% variance in tourism across the countries.

**Fig 4:**
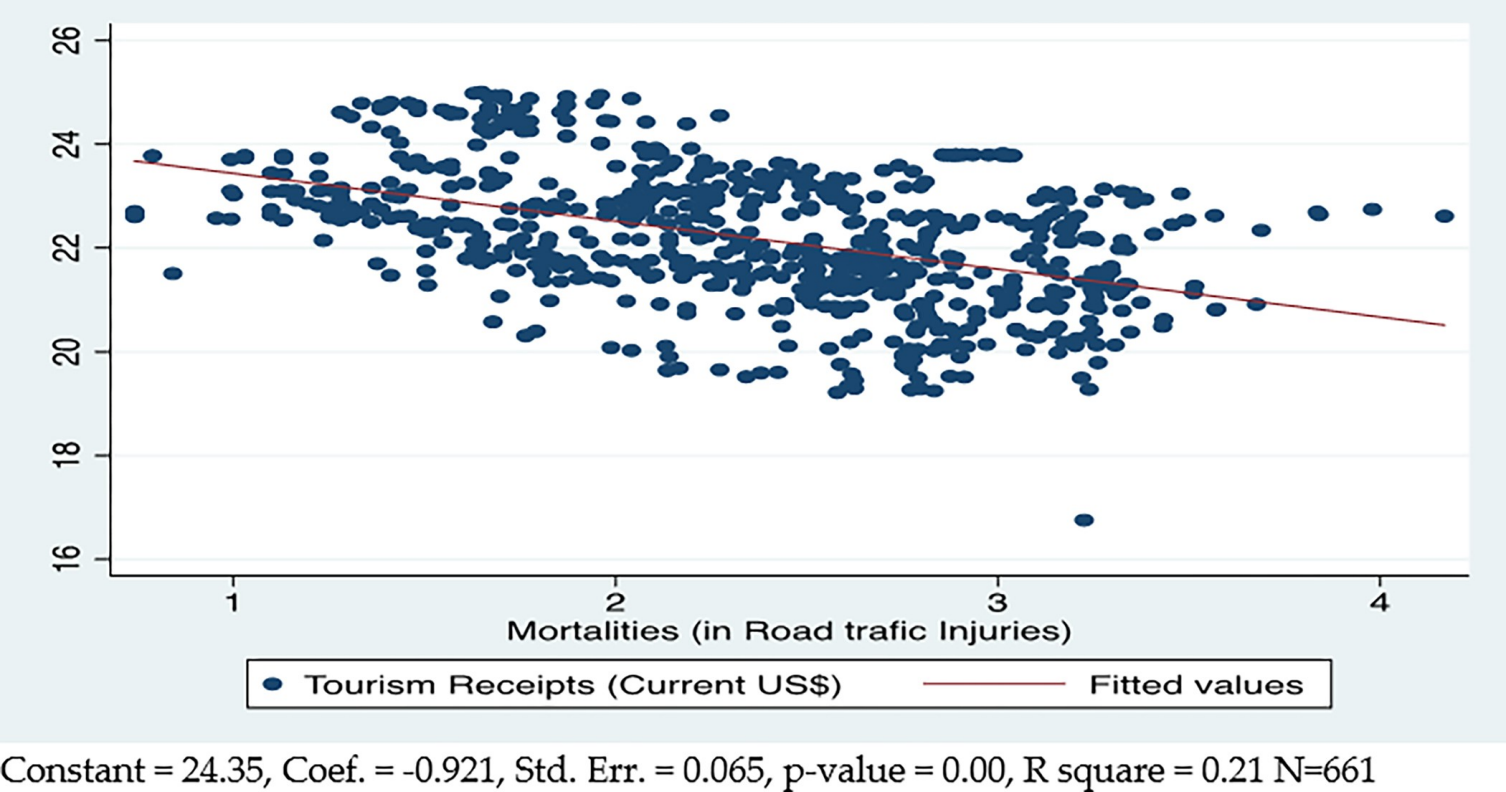
Mortality from road injuries and tourism.

Fig 4 shows that there is a negative and significant relationship between deaths caused by road accidents and tourism. It states that a 1% increase in road fatalities will result in a 0.92% decrease in tourism. The fatalities due to road injuries explain the 21% variance in tourism.

**Fig 5:**
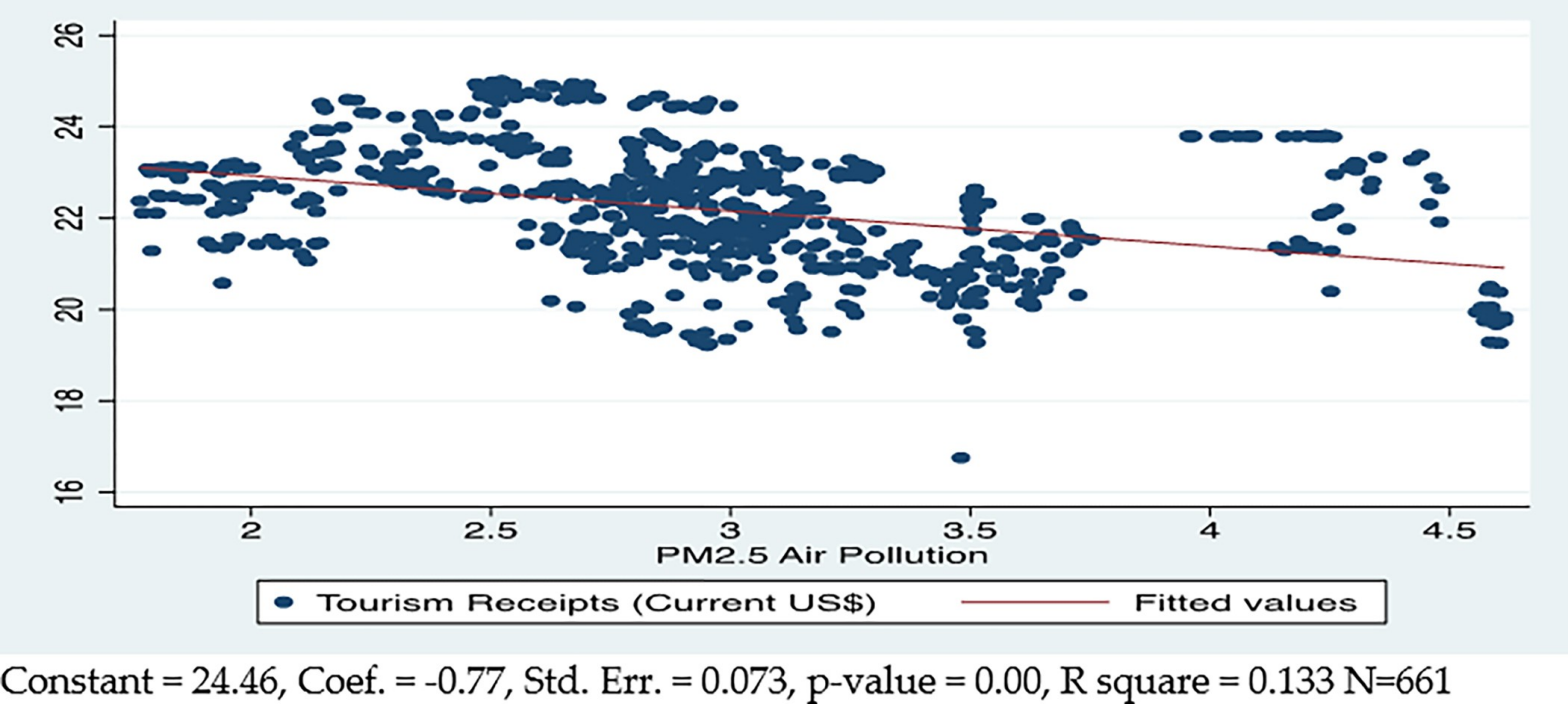
PM 2.5 (air quality) and tourism.

Fig 5 plots the aggregate correlational linear relationship between the air quality (PM 2.5) and tourism for the sampled countries, which provided the preliminary estimates for the existence of the relationship between these variables. The results depicted that bad air quality has a significant negative relationship with tourism; a 1% decrease in quantity of PM 2.5 causes a 0.77% increase in country tourism. It explains the 13% cross-country variance in tourism.

**Fig 6:**
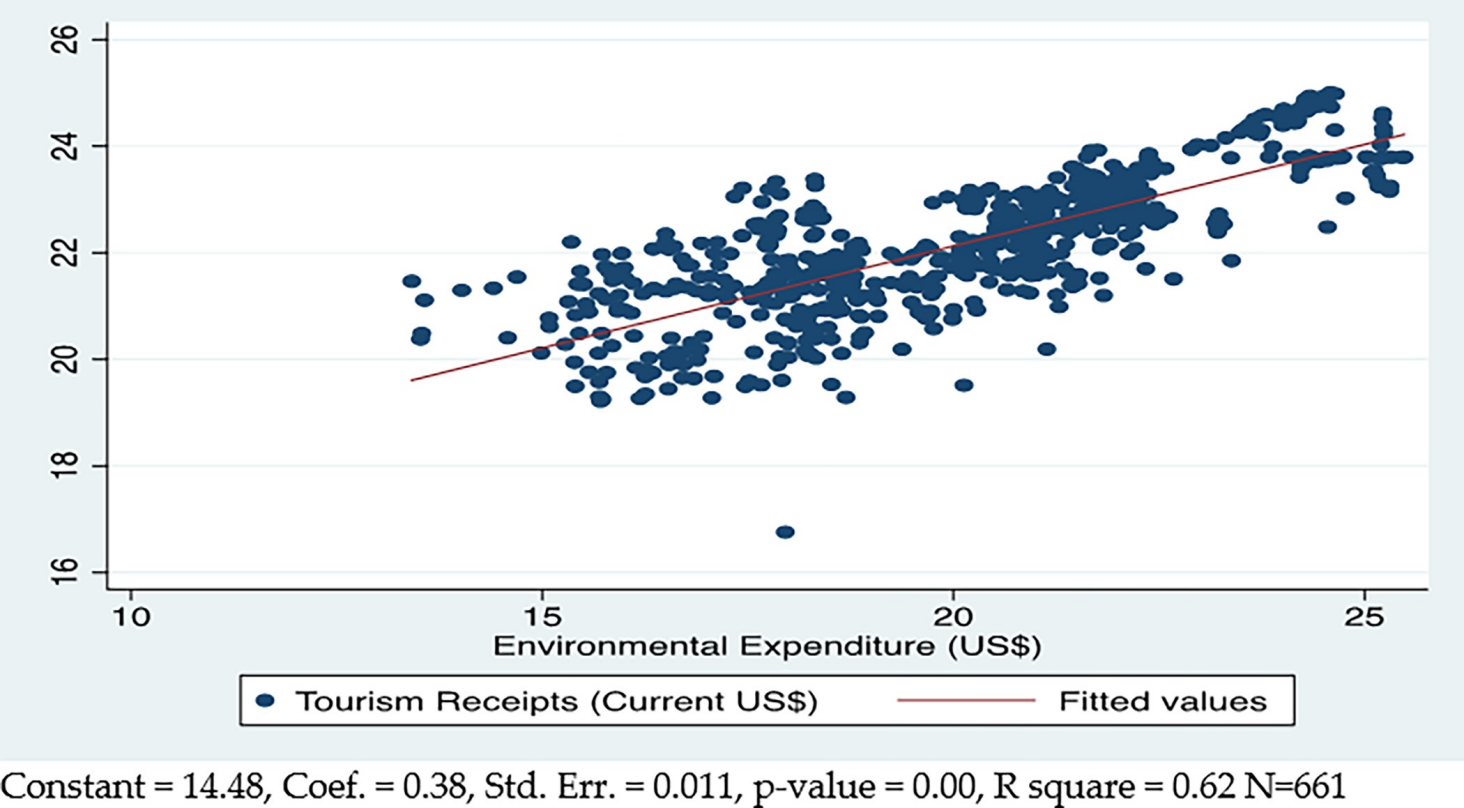
Green finance and tourism.

The green finance improves the tourism of the countries, which can be described by the results available in Fig 6. The aggregate linear correlation between green finance and tourism is positive and significant at 1%. It means the 1% increase in green finance will increase inter-country tourism by 0.38%. This tends to explain the 62% variance in tourism between countries.

### 4.3. Empirical model results

The short run and long run coefficients estimated by using SYS-GMM are given in Table 3. The coefficient of the lagged variable of tourism indicates that international tourism has a significant effect on achieving sustainable tourism development. In the short run, the health disasters and crises were negatively and significantly affecting tourism. The results regarding deaths due to health disasters and crises indicate that the 1% increase in deaths will reduce tourism by 0.07%. Similarly, in the long run, the coefficient of health disasters and crises is also significant at 5%, which explains that a 1% increase in deaths will reduce international tourism by 0.02% in the long run. The number of deaths per year has a smaller negative impact on tourism In the long run than in the short run. In both the short and long run, the quality of the health system has a significant positive effect on international tourism. In the short run, a 1% growth in health expenditures will increase tourism by 0.23%, and in the long run, a 1% rise in health expenditures will increase international tourism by 1.1%. The long-term impact of health system quality outweighs the short-term impact. Furthermore, the relationship between health expenditure and international tourism is inelastic in the short run but elastic in the long run.

**Table 3:** Results of SYS-GMM model.

Variables	SYS-GMM	
	Short run coefficient	Long run coefficient
T-l1	0.79* (0.06)	---
Health system quality	0.23* (0.08)	1.1* (0.41)
Transportation carbon footprints	-0.32*** (0.18)	-1.51*** (0.88)
Mortality from road injuries	-0.18*** (0.09)	-0.86 (0.58)
Health disasters and crises	-0.07* (0.01)	-0.02** (0.009)
Air quality	-0.22** (0.10)	-1.04*** (0.57)
Green finance	0.1** (0.04)	0.46*** (0.25)
Years dummies	Yes	
No. Of observations	661	
F statistics	153539.64	
Groups/instruments	51/42	
Ar (2)	0.214	
Hansen statistics	0.236	

Notes: *,**,*** are statistical significance at 1%, 5% and 10% levels respectively. Values in parentheses are standard errors.

The results also showed that the quality of the transportation infrastructure (transportation footprints and mortality from road injuries) negatively influenced tourism. The transportation carbon footprints also have an inelastic short-run effect on tourism. In the short run, a 1% increase in the CO2 emissions from transportation will reduce tourism by 0.32%. In the long run, a 1% increase in CO2 emissions from transportation will decrease international tourism by 1.51%. In the long run, the relationship between emissions from transportation and tourism is elastic. A 1% increase in mortalities due to road injuries will reduce international tourism by 0.18% in the short run, while it has no significant relationship with tourism in the long run. However, mortality due to road injuries and tourism has an inelastic relationship in the short run.

The model results for air quality show that a 1% increase in PM 2.5 causes a 0.22% decrease in international tourism, indicating an inelastic relationship between air quality and tourism in the short run. On the other hand, a 1% increase in PM 2.5 will decrease international tourism by 1.04%. This demonstrates that air quality and tourism have a greater long-term impact than a short-term one.

Green financing has a significant short- and long-term impact on international tourism. In the short run, a 1% increase in green finance will cause 0.10% increase in tourism. In the long run, the effect of green finance on tourism is larger than in the short run because a 1% increase in green finance will increase tourism by 0.46%.

### 4.4. COVID-19 and tourism

**Fig 7:**
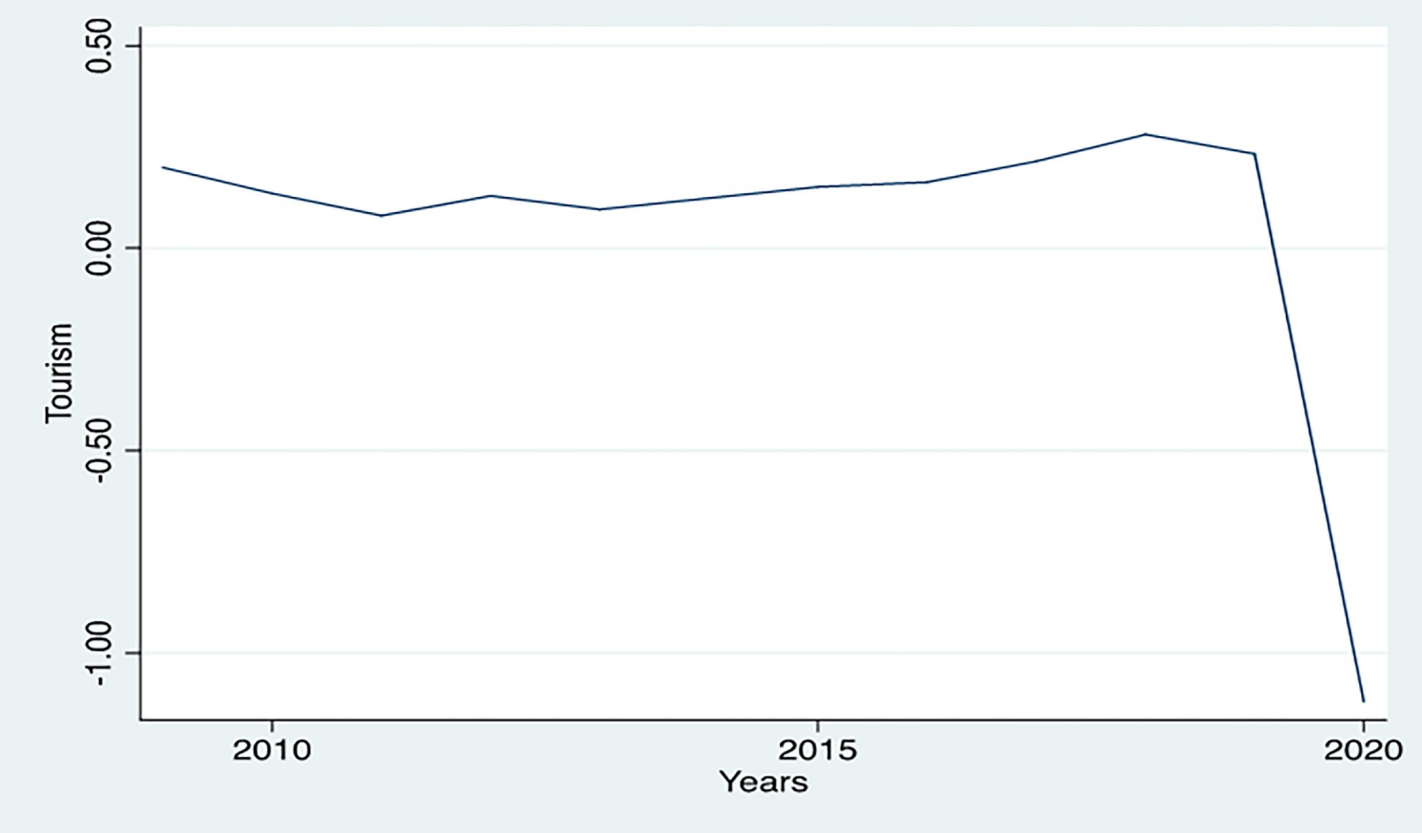
Tourism trends (2007–2020).

The Fig 7 describes the estimated fluctuation in tourism over the years in the sampled countries. It shows that tourism had been growing prior to COVID-19. The coefficients of the years were plotted, and the change in tourism over the years relative to the base year (2007) was calculated using (eyears’ coefficient (B)- 1)*100. The results show that in 2019, tourism was slightly higher than in base years. It means that tourism in 2019 was 26.24% higher than in 2007. Following the declaration of COVID-19 as a health emergency, the world imposed strict mobility restrictions due to the global health crisis, resulting in a 67.37% decrease in tourism in comparison to 2007.

### 4.5. Robustness

We looked at the impact of various additional control variables on the findings during the robustness check. We used the log of urban population as a percentage of total population, and the log of population density because these variables have a significant correlation with tourism [42]. Table 4 describes the results regarding the findings of the current experiment. The main explanatory variables did not show any shift in signs or significance.

**Table 4:** Robustness check results.

Variables	SYS-GMM
T-l1	0.59* (0.04)
Health system quality	0.13* (0.02)
Transportation carbon footprints	-0.27** (0.12)
Mortality from road injuries	-0.11*** (0.06)
Health disasters and crises	-0.04* (0.01)
Air quality	-0.12** (0.06)
Green finance	0.03** (0.01)
Variables	SYS.GMM.

Notes: *,**,*** are statistical significance at 1%, 5% and 10% levels respectively. Values in parentheses are standard errors.

## 5. Discussion

Tourism is an important sector for world economic growth and requires special attention and different policies during various health disasters and crises for sustainable tourism. The panel of 51 countries in this study is subjected to the six different repressors in this study (health disasters and crises, quality of health systems, transportation carbon footprints, mortality from road accidents, air quality, and green finance). The lagged variable for tourism in the current study is positive and significant. More international tourism in a year contributes positively to the development of tourist places, which attract more tourists the following year. The increase in revenue at the tourist sites induced the development activities to proceed at a high pace. This means that tourism contributes to sustainable tourism and development. These results are in line with Gao et al. [42].

Health disasters and crises are also one of the most important factors that affect the international tourism inflow. Health disasters and crises both result in high death rates, influencing tourists’ perceptions and behaviors toward the touring country. The world has become a global village, and people are inextricably linked to one another. News of various disasters and crises reaches every part of the world quickly, both via social and mainstream media, ultimately influencing the decisions of foreign tourists. The media fuels such factors by creating a bad image of the destination [78], which significantly affects the tourist’s decision and their choice of destination due to health and safety concerns and personal security perceptions of tourists [79,80]. The health-related crises are highly linked to the negative coverage and graphic imagery of the media, which make the tourism sector challenging to manage [81]. As a result, tourism is one of the most vulnerable industries, vulnerable to crises and disasters [82,83]. The countries’ health crises increase deaths [84] and have a direct negative impact on tourism [85]. The current study also found the negative impact of deaths on international tourism, which describes the negative impact of the health crises on tourism. Due to the popular media, the issue of deaths in tourism has sparked public interest and drawn the attention of academicians and scholars [86]. Without a doubt, the diseases cause a large number of deaths or prolonged illnesses when traveling abroad or returning home [87]. Furthermore, many previous studies have investigated the epidemic’s impact on tourism flows [88]. Foot and mouth disease outbreaks in the UK in 2001 decreased tourist inflow by 9%, and could not return to the normal level until 2003 [89]. Similarly, the SARS epidemic in China had an impact on the country’s tourism [90]. The Ebola epidemic in Africa also negatively affected tourism [91]. Similarly, Gaoe et al. [42] also demonstrated that tourism declines as health-related crises exist at the destination.

Ecofriendly and safe transportation is a basic necessity and a prerequisite for sustainable tourism in any country [92]. Better transport facilities and road safety improve tourist perceptions of visiting the attractions or destinations [87]. The current study also found the negative impact of mortality due to road injuries on international tourism, which describes how safe transport and road infrastructure promote international tourism. Transportation and roads play an important role in the development of tourism because they connect people and places [93]. An efficient transport system and road infrastructure reduces trauma and injuries during travel. The low injuries and proper and early first aid on the spot cause few deaths, which also attract international tourism. Mohammed et al. [94] described the developing countries as more dangerous in terms of road accidents due to the poorly managed traffic system, low standards of safety, and low public awareness. Hence, the lack of basic road infrastructure makes the tours more risky, unsafe, and uninteresting, which results in unpleasant tourism services that cause a low inflow of tourism [95]. Moreover, a healthier and wealthier region is a strike necessity for sustainable tourism [96]. Literature has well explained the relationship between tourism indicators and health expenditures. For example, Li et al. [97] in Malaysia, Kumar et al. [98], and Deskins and Seevers [99] in the United States focused on health tourism and tourism expenditures. Health system quality depicts the well-managed and well-facilitated health system of the country. Our results are in line with those of Ozturk [100] and Cheah and Abdul-Rahim [101], who also reported a positive and significant, impact of health expenditures per capita on tourism. Lee [102] also found a long-term positive relationship between health care and international tourism.

The current study found both short- and long-term negative impacts of poor air quality on international tourism. The results related to air quality corroborate with Zhou et al. [103] who also reported a negative impact of low air quality on tourism. Li et al. [104] also found a considerable effect of PM2.5 on tourism in China. Moreover, Zhang et al. [53] and Becken et al. [54] also described the importance of air quality on tourism. According to Li [105], there is growing awareness and concern regarding the state of the environment in China, specifically the inadequate air quality observed in its major cities. This issue has had a detrimental impact on China’s appeal as a destination for travelers and its efforts to position itself as a sustainable tourist destination. Individuals have voiced skepticism regarding the adverse impact of air quality and pollution on tourist arrivals. Transportation has a major share in world carbon footprints and in degrading air quality, which, according to the current study, have a negative impact on international tourism. This shows that ecofriendly transportation system can positively affect the sustainable tourism.

Green finance includes the financial activities that have been conducted to ensure a better environmental outcome. The findings of green finance on tourism are consistent with previous research from Ip et al. [22], Zhang [106], and Xiugang [107] who have also described the positive impact of green finance on tourism. The findings related to the impact of COVID-19 on tourism are in line with Gao et al. [26] who also stated that COVID-19 substantially reduced international tourism.

## 6. Conclusions

Tourism is one of the main sectors that contribute significantly to sustainable economic development, but the tourism industry in any country is influenced by a number of factors. The significant negative impact of health crises and disasters implies that a 1% increase in deaths will reduce tourism by 0.07% in the short term and 0.02% in the long term. The 1% increase in CO2 emissions from transportation reduces tourism by 0.32% in the short run, while it has a significant impact in the long run by reducing tourism by 1.51%. Mortalities due to road injuries decrease tourism by 0.185 in the short run and 0.86% in the long run. The air quality impact was elastic in the long run, with a coefficient value equal to 1.04%, and inelastic in the short run, with a coefficient value of 0.22%. The significant positive impact of green finance implies that tourism will increase by 0.10% in the short run and by 0.46% in the long run due to a 1% increase in environmental protection expenditure. The model results depicted that health disaster and crises, the quality of health systems, transportation carbon footprints, and mortality from road accidents, air quality, and green finance all significantly affect international tourism. This demonstrates that the presence of a well-managed health system, an environmentally friendly and safe transportation system, better air quality, and more green finance in terms of pollution reduction investment increase international tourism in the destination country. To accomplish this, financial institutions should accelerate their policies to provide cheaper green loans in order to improve investment in reducing environmental pollution. Further, green finance experts and financial authorities view the involvement of environmental protection departments in the development and enforcement of regulations at various tourist destinations as essential. Governments must ensure harmonious coordination between monetary and fiscal policies. To enhance the ecological conditions of tourist destinations and attract visitors, it is imperative to strategically adjust the financial allocations and government expenditures related to controlling environmental pollution. This approach aims to achieve mutually beneficial outcomes for both the environment and the tourism industry.

The main limitation of this study was the availability of data for all countries. The limited availability of data for the countries reduced the number of countries to 51 in this study. Moreover, the study does not take into account the demographic differences among countries for data analysis, which might have affected the results of the study. There are some other variables that may affect sustainable tourism during the pandemic, such as digitalization, but these could not be considered due to the unavailability of authentic data for the studied countries. Despite these limitations, this study provides insightful findings for policymakers aimed at sustainable tourism worldwide.

## Supporting information

S1 File (RAR)
